# Innovation in Laparoscopic Inguinal Hernia Reparation – Initial Experiences with the Parietex Progrip Laparoscopic^™^ – Mesh

**DOI:** 10.3389/fsurg.2015.00028

**Published:** 2015-06-25

**Authors:** Pavol Klobusicky, Peter Feyerherd

**Affiliations:** ^1^Department of General and Digestive Surgery, Helios St. Elisabeth Hospital Bad Kissingen, Bad Kissingen, Germany

**Keywords:** laparoscopic hernia repair, self-adhesive mesh, TAPP

## Abstract

**Introduction:**

The Laparoscopic TAPP (transabdominal pre-peritoneal inguinal hernia repair) – technique is becoming more widely and frequently used due to higher patient satisfaction and lower rates of both relapse and complications.

**Materials and methods:**

The role of the fixation of the mesh is especially important in regard to the endoscopic technique. The fixation of mesh through penetrating techniques using staples, clips, or screws is associated with a significantly increased risk of developing a chronic post-herniotomy inguinal pain syndrome (CPIP). In order to answer the question “fixation or no fixation of the mesh,” the use of self-adhesive mesh is an optimal compromise.

**Conclusion:**

With the authors own operative technique consisting also of a standard pre- and post-operative management, the self-adhesive mesh was proven to be extremely reliable. As no specific materials to fix the mesh were needed, the method was fast, simple, and economical. We could also reduce the incidences of chronic inguinal pain in our patient population by using the self-adhesive mesh, without the risk of an increased recurrence rate in the observation period.

## Introduction

Inguinal hernia repair is one of the most frequently performed surgical procedures worldwide in general surgery. There are approximately 700,000 hernia repairs performed in the whole world every year, which results in costs of approximately USD 500,000,000 in the United States plus the costs for medication, sickness leave, and missing work performance. According to the German DRG system, the costs for surgical therapy of inguinal hernia are around 322,000,000 Euro. This means that from the point of costs – an effective and reliable therapy of inguinal hernia is a significant factor that influences efficiency of national systems of public health insurance (1, 2). In the last 20 years, open inguinal hernia repair, which uses a technique first described by Lichtenstein, has become the Golden Standard. However, if you take “Guidelines for laparoscopic and endoscopic care of inguinal hernia” into consideration, which was published in 2011, an increasing presence of minimally invasive procedures can be found in the field of inguinal hernia care (3). Transabdominal laparoscopic (TAPP) approach in the therapy of inguinal hernia seems to be a suitable alternative to classical open inguinal hernia repair, mainly in the hands of an experienced surgeon (4). According to several studies comparing open and endoscopic/laparoscopic hernia repair is minimal, invasive inguinal hernia repair is a suitable alternative, mainly in case of recurrent hernias and bilateral hernias (1, 3, 5–7). Numerous studies have now shown that the open technique and the endoscopic technique are procedures that are quite comparable, although the laparoscopic techniques offer advantages in certain respects, particularly in the hands of an experienced surgeon (5, 8). Laparoscopic inguinal hernia repair offers the possibility of gentle dissection with posterior implantation of the mesh and possibility of minimal invasive fixation of implanted mesh (2, 8–10).

On the market, there were several prosthetic materials introduced that were intended for implantation to the groin, the safety and efficacy of which was demonstrated by several studies (7). Originally, it was necessary and recommended to stabilize the implant at the site of implantation with additional invasive fixation in a form of spirals, clips, screws etc. These implants in inappropriate localization were often the cause of CPIP and also relatively dramatically increased the costs for that surgery. This resulted in increase of attempts to minimize invasive fixation of implanted mesh with the use of tissue glue, absorbable fixation materials, and self-adhesive meshes. The option to use self-fixation meshes in inguinal hernia repair was verified on an animal experiment as safe, well tolerated, simple to perform with good macroscopic and microscopic integration to the abdominal wall (11). During further course, there were several self-fixation meshes introduced to the market that were intended for open and endoscopic implantation (12).

## Materials and Methods

The Laparoscopic TAPP (transabdominal pre-peritoneal repair) – and the endoscopic TEP (total extra-peritoneal repair) – technique is becoming more widely and frequently used due to higher patient satisfaction and lower rates of both relapse and complications, and therefore the medical industry is increasingly focusing on products in inguinal hernia repair in order to steadily improve the user-friendliness for the surgeon and the safety of the patient. The role of the fixation of the mesh is especially important in regard to the endoscopic technique (1, 2).

A fixation of mesh is avoidable in certain situations, especially when using the TEP-technique (13). However, the fixation of mesh through penetrating techniques using staples, clips, or screws is associated with a significantly increased risk of developing a chronic post-herniotomy inguinal pain syndrome (CPIP). Alternative fixation techniques such as the use of different adhesives are, however, associated with increased costs. In order to answer the question “fixation or no fixation of the mesh,” the use of self-adhesive mesh is an optimal compromise.

Ever since a new, self-adhesive mesh, Parietex Progrip™, first in the USA and also later in Europe, was launched, the mesh has become increasingly popular and now has a stable place both in the open care of inguinal hernia and in endoscopic procedures. It is a lightweight, self-adhesive mesh consisting of monofilament polyester and polylactic acid (PLA), which was originally only developed and intended for the use in the Lichtenstein-technique. As recent studies have proven, the use of the Parietex Progrip TM mesh is accompanied by a significant reduction of, both, cost and surgery time (14). Although the implantation of the Parietex Progrip™ mesh as part of the endoscopic techniques is technically challenging, it can yet be feasible in experienced hands (15). The Parietex Progrip™ mesh has been applied successfully in our clinic for TAPP-hernioplasty since 2008 and we can boast about executing more than 500 Hernia-Repairs using this particular technique. Since this mesh was not originally intended for use in the field of minimally invasive hernioplasty, the usage initially caused some difficulties and needed for the mesh to be specially prepared before using this specifically developed implantation technique. Thanks to the rapid dissemination of this method and the more frequent using of the self-adhesive mesh, especially with TAPP-, but also with the TEP hernioplasty, the enhanced Perietex Progrip Laproscopic™ mesh has been released. Parietex Progrip Laparoscopic™ (Sofradim Production, Covidien) is a self-gripping composite mesh, consistent of a monofilament polyethylene terephthalate (PET) mesh, covered by a resorbable layer of microgrips made of monofilament PLA combined with segmental covering of Fast Resorbing Film Composition (70% Collagen, 30% Glycerol). Weight of the mesh before absorption is 82 g/m^2^ and weight after absorption is 49 g/m^2^ with the Pore Size (millimeter) 1.8 × 1.8 (macroporous). According to the manufacturer is the degradation of the quickly absorbing layer <1 day and the layer with microgrips >18 months. Only since then has the technically more sophisticated and elaborate implantation of the mesh become a procedure that is simple and easier to learn.

## Surgical Technique

**Figure 1:**
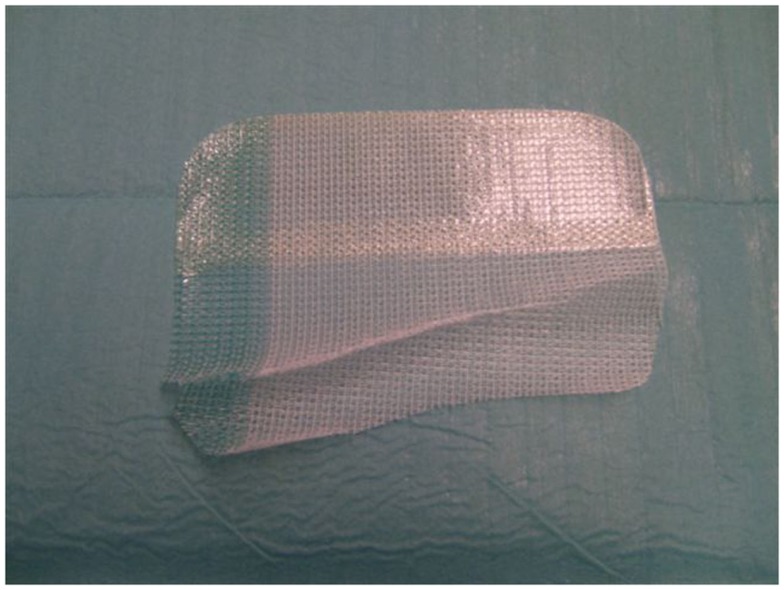
**Parietex Progrip Laparoscopic™ mesh with visible coating on the upper part and self-adhesive on the lower section of the mesh**. The medial side of the net is marked green. Clearly recognizable anatomical shape of the mesh with separate alignments for the right and the left groin.

**Figure 2:**
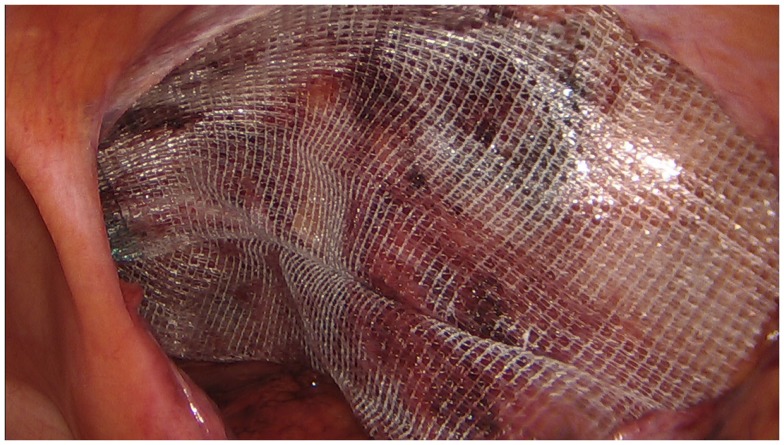
**Summary view of the implanted net**. Optimal position of the implant with sufficient overlap of all potential defects.

Preparation of the inguinal hernia using the standardized technique. Standard intravenous anesthesia with orotracheal intubation that enables Trendelenburg position. Operation was performed using 3 trocars, 12 mm trocar above umbilicus and 5 mm + 12 mm trocars at the level of the umbilicus. A 30° optics is used. After accessing, the inguinal region is performed dissection of parietal peritoneum on the affected side at the direction from spina iliaca, anterior superior up to plica umbilicalis medialis at the same side. During dissection are visualized and prepared gonadal vessels, vas deferens, or the round ligament (in women), Cooper’s ligament, and the posterior fascia of the rectus abdominis muscle. After termination of dissection in the groin, we introduced prepared implant through 12 mm trocar. In the abdominal cavity is the implant spreaded and placed to the groin in order to cover hernia opening of at least 2–3 cm to all directions and also to cover other preformed weaker sites in the groin. Fixation to abdominal wall is accomplished with gentle pressing the implant against the abdominal wall using a surgical instrument, best with a tampon. Fixation occurs based on a mechanical effect by adherence of grips to tissue (Figures 1 and 2). Then follows reconstruction of parietal peritoneum with continuous absorbable suture. In case of a bilateral hernia, follows its treatment with the same method and technique with covering both meshes in pre-vesical space without the need to change the location of trocars. To reduce urethral manipulation in an effort to minimize post-operative urinary retention, patients void pre-operatively and a Foley catheter is not routinely placed. No antibiotic prophylaxis is routinely administered during the surgery.

## Discussion

Laparoscopic inguinal hernia repair became one of the standard methods to treat inguinal hernia, mainly in cases of recurrent hernias, bilateral hernia, and femoral hernia (16). More than 70 different studies and meta-analyses comparing laparoscopic endoscopic and open inguinal hernia repairs stated comparable long term results focusing on recurrences, post-operative pain, and QoL of patients (1, 3, 5, 6). Mild differences were reported in the occurrence of chronic post-operative pain (3). One of the remaining problems to treat in the field of laparoscopic inguinal hernia repair remains the problem of fixation or non-fixation of prosthetic material. It has been shown that traumatic fixation of mesh increases the possibility to develop CPIP. This is the reason why atraumatic fixation of prosthetic material is recommended (9). For example in case of endoscopic TEP, technique has been shown that it is possible to withdraw from traumatic fixation of the implant, without increased risk of recurrences (3). In case of laparoscopic inguinal hernia repairs was shown efficiency of so called non-traumatic fixation of the implant, e.g., with tissue glue without increased recurrence rate. Alternative to atraumatic fixation with tissue glues is usage of so called self-fixation mesh, which enables sufficient implant fixation to the abdominal wall, provided by specially adjusted surface without the need of additional fixation (2, 4).

The Parietex Progrip Laparoscopic mesh provides a laparoscopically oriented surgeon with an optimized mesh for both laparoscopic and endoscopic inguinal hernia care. Due to its anatomical shape, anti-adhesive partial coating, and separate left and right alignment, even surgeons that are less-experienced using self-adhesive meshes can quickly and easily use this user-friendly mesh. The relatively higher price of the implant appears to be slightly disadvantageous at first, but since the implant does not require any additional fixation, the satisfaction and the safety of the patient, especially in respect to post-operative chronic pain, can be increased, keeping the relapse rate low.

## Conflict of Interest Statement

The authors declare that the research was conducted in the absence of any commercial or financial relationships that could be construed as a potential conflict of interest.
